# Dataset of the transcribed 45S ribosomal RNA sequence of the tree crop “yerba mate”

**DOI:** 10.1016/j.dib.2017.04.044

**Published:** 2017-05-06

**Authors:** Patricia M. Aguilera, Humberto J. Debat, Mauro Grabiele

**Affiliations:** a Instituto de Biología Subtropical, Universidad Nacional de Misiones (CONICET), 3300 Posadas, Misiones, Argentina; b Instituto de Biotecnología de Misiones, Universidad Nacional de Misiones, 3300 Posadas, Misiones, Argentina; c Instituto de Patología Vegetal, Centro de Investigaciones Agropecuarias (INTA), 5000 Córdoba, Argentina

**Keywords:** Ilex, Transcriptomics, Assembly, Annotation, RNA unit

## Abstract

This contribution contains data related to the research article entitled “The 18S-25S ribosomal RNA unit of yerba mate (*Ilex paraguariensis* A. St.-Hil.)” (Aguilera et al., 2016) [1]. Through a bioinformatic approach involving NGS data, we provide information of the transcribed 45S ribosomal RNA (rRNA) sequence of yerba mate, the first reference for the *Ilex* L. genus. This dataset (Supplementary file 1) comprises information regarding the assembly and annotation of this rRNA unit. The generated data is applicable for comparative analysis and evolutionary studies among *Ilex* and related taxa. The raw sequencing data used here is available at DDBJ/EMBL/GenBank (NCBI Resource Coordinators, 2016) [2] Sequence Read Archive (SRA) under the accession SRP043293 and the consensus 45S ribosomal RNA sequence has been deposited there under the accession GFHV00000000.

**Specifications Table**

**Table:** 

Subject area	Biology
More specific subject area	Evolutionary biology
Type of data	Transcriptomics, assembly of reads and sequence annotation
How data was acquired	Bioinformatics approach
Data format	Analyzed
Experimental factors	RNA, used for library construction and Illumina sequencing, was isolated from leaf samples at emerging, young, fully expanded, and early and late senescent stages from *I. paraguariensis* breeding line Pg538
Experimental features	Paired-end 100 nt Raw reads were filtered, *de novo* assembled and submitted to homology-based searches and annotation
Data source location	Misiones, Argentina
Data accessibility	Data are within this article and at DDBJ/EMBL/GenBank under the accessions SRP043293 and GFHV00000000

**Value of the data**

• This data provides the first reference sequence of the transcribed 45S rRNA unit in *Ilex* L.
• Data is applicable for comparative analysis and evolutionary studies among *Ilex* and related taxa based on the 18S, 5.8S and 25S rRNA genes and ITS and ETS sequences.
• Accessibility of assembly and annotation data allows researchers to perform further analysis via novel approaches.

## 1 Data, experimental design, materials and methods

Total RNA extracted of five samples of emerging, young, fully expanded, and early and late senescent stages leaves of *Ilex paraguariensis* breeding line Pg538 were pooled for high throughput sequencing [1].

The attainment of the transcribed 45S rRNA sequence of yerba mate was completed in seven steps:

1. The complete raw sequencing data at SRA under the accession SRP043293 was used to generate a full transcriptome assembly employing the Trinity 2.0.6 platform. All raw sequenced reads were quality filtered and then *de novo* assembled into contigs with optimal parameters of 25 kmer word and group pairs distance of 500.
2. The achieved complete list of 44,907 contigs was subsequently scanned by in-house [3; v.8.1.8] homology searches with BLASTN (word size 11, cut off value of 1e-10) using as baits the conserved 18S, 5.8S and 25S rRNA entire gene regions of *Helianthus annuus* 45S (KF767534). Sunflower was selected, as it is the closest taxon to yerba mate in Euasterids II clade [4] from which complete rRNA sequence information is available yet.
3. Three blast hits were obtained and identified as contigs comp17895_c0_seq. 1 (2936 bp), comp17895_c1_seq. 1 (1053 bp) and comp17901_c0_seq. 2 (2994 bp), which assemble in a 6961 bp sequence (Supplementary Figure 1, BAM file).
4. This sequence was further aligned to the sunflower rRNA reference sequence regions [3] following the methods of Geneious global alignment with free end gaps (93%, gap open penalty 12, gap extension penalty 3) and progressive Mauve algorithm [5] at default values. The alignments were manually checked previous to the transference of homology-based annotations among sunflower and yerba mate.
5. The annotated sequence which in turns spans the complete 45S rRNA unit of yerba mate, shares a consistent pairwise similarity of 54.2%, 97.6%, 54.3%, 96.2%, 66.4%, 95.7% and 49.7% at the 5´ETS, 18S, ITS1, 5.8S, ITS2, 25S and 3´ETS, respectively, with sunflower.
6. This first annotation step of the yerba mate rRNA sequence was further curated by subsequent homology searches [3] in non-redundant NCBI *Ilex* databases by BLASTN (word size 11, cut off value of 1e-10). Supplementary Figures 2–6 (BAM files) illustrate this step for each ribosomal RNA region with blast hits, and the accession of hits and coverage are provided.
7. A final yerba mate annotated ribosomal RNA sequence embracing genes (18S, 5.8S, 25S) and spacers (ITS1, ITS2, 5´ETS, 3´ETS) was attained by last integration of sunflower and *Ilex* annotated features. Suplementary Figure 7 (GFF file) illustrates this final step.

This Transcriptome Shotgun Assembly project has been deposited at DDBJ/EMBL/GenBank [2] under the accession GFHV00000000. The version described in this paper is the first version, GFHV01000000.

## Appendix A Supplementary material

Suppl. Fig. 1. Assembled 45S rRNA unit of yerba mate.

.

Suppl. Fig. 2. 18S rRNA region of yerba mate and associated Ilex NCBI blast hits.

.

Suppl. Fig. 3. 25S rRNA region of yerba mate and associated Ilex NCBI blast hits.

.

Suppl. Fig. 4. 5.8S rRNA region of yerba mate and associated Ilex NCBI blast hits.

.

Suppl. Fig. 5. ITS1 rRNA region of yerba mate and associated Ilex NCBI blast hits.

.

Suppl. Fig. 6. ITS2 rRNA region of yerba mate and associated Ilex NCBI blast hits.

.

Suppl. Fig. 7. Annotated 45S rRNA unit of yerba mate.
