# Supplementary material for: Dataset of the transcribed 45S ribosomal RNA sequence of the tree crop “yerba mate”
Source: Data Brief. 2017 May 6;12:649–51. doi: 10.1016/j.dib.2017.04.044 (PMC5432670; doi:10.1016/j.dib.2017.04.044)
Supplement: Supplementary file 1 — Supplementary material [file mmc1.docx]

**Conflict of Interest Form.**

Authors declare that there is no conflict of interest.

Patricia Aguilera and colleagues.
